# Selective Induction of Human Autonomic Neurons Enables Precise Control of Cardiomyocyte Beating

**DOI:** 10.1038/s41598-020-66303-3

**Published:** 2020-06-11

**Authors:** Yuzo Takayama, Hiroko Kushige, Yuka Akagi, Yutaka Suzuki, Yutaro Kumagai, Yasuyuki S. Kida

**Affiliations:** 1 Cellular and Molecular Biotechnology Research Institute, National Institute of Advanced Industrial Science and Technology (AIST), Central 5-41, Higashi 1-1-1, Tsukuba, 305-8565 Ibaraki Japan; 2 Advanced Photonics and Biosensing Open Innovation Laboratory, National Institute of Advanced Industrial Science and Technology (AIST), Central 5-41, Higashi 1-1-1, Tsukuba, 305-8565 Ibaraki Japan; 3 Department of Computational Biology and Medical Sciences, The University of Tokyo, 5-1-5 Kashiwanoha, Kashiwa-shi 277-8562 Chiba, Japan

**Keywords:** Stem-cell biotechnology, Autonomic nervous system, Pluripotent stem cells, Stem-cell differentiation

## Abstract

The autonomic nervous system (ANS) regulates tissue homeostasis and remodelling through antagonistic effects of noradrenergic sympathetic and cholinergic parasympathetic signalling. Despite numerous reports on the induction of sympathetic neurons from human pluripotent stem cells (hPSCs), no induction methods have effectively derived cholinergic parasympathetic neurons from hPSCs. Considering the antagonistic effects of noradrenergic and cholinergic inputs on target organs, both sympathetic and parasympathetic neurons are expected to be induced. This study aimed to develop a stepwise chemical induction method to induce sympathetic-like and parasympathetic-like ANS neurons. Autonomic specification was achieved through restricting signals inducing sensory or enteric neurogenesis and activating bone morphogenetic protein (BMP) signals. Global mRNA expression analyses after stepwise induction, including single-cell RNA-seq analysis of induced neurons and functional assays revealed that each induced sympathetic-like or parasympathetic-like neuron acquired pharmacological and electrophysiological functional properties with distinct marker expression. Further, we identified selective induction methods using appropriate seeding cell densities and neurotrophic factor concentrations. Neurons were individually induced, facilitating the regulation of the beating rates of hiPSC-derived cardiomyocytes in an antagonistic manner. The induction methods yield specific neuron types, and their influence on various tissues can be studied by co-cultured assays.

## Introduction

Biological data and animal studies have provide a basis for medical research and drug discovery. However, recent data from animal studies have poorly recapitulated human characteristics owing to inter-species genetic variations^1,2^. Accordingly, numerous attempts to develop *in vitro* assays, including cell-based assays and device-based co-culture assays using human cells and tissues, have attempted to replace animal models and model human physiological conditions.

Devices often include microfluidic structures for mimicking inter-organ communication in blood flow, including physiological nutrition and biomolecular gradients^3^. Furthermore, the peripheral nervous system (PNS) regulated organ functions and transmits signals originating in the central nervous system to organs. However, co-culture systems containing PNS innervations are not well characterized.

The PNS contains sensory, enteric, sympathetic, and parasympathetic neurons, which are surrounded by peripheral glial cells and connective tissues. Sympathetic and parasympathetic neurons, constituting the autonomic nervous system (ANS), regulate organ/tissue homeostasis through opposite effects. Consequently, inherited and acquired ANS disorders, including familial dysautonomia^4^ and diabetic neuropathy^5^, cause various systemic symptoms, including respiratory failure, abnormal blood pressure, and irregular heart rate. Furthermore, the ANS contributes to organ development^6^ and tumour progression^7^ via neuronal signal-mediated cell survival and migration. Thus, *in vitro* derivation of both ANS neurons from human pluripotent stem cells (hPSCs) would help model human physiological conditions and ANS-related disorders.

Each type of PNS neuron is derived from different neural crest (NC) cells that migrate from various regions along the anterior-posterior axis. Therefore, previous studies attempting to generate PNS neurons from hPSCs initially induced corresponding NC cells and differentiated them into neurons. Along with recent technical advancements in stem cell biotechnology, these studies have derived sensory neurons^8^, enteric neurons^9^, and sympathetic neurons^10–12^ from hPSCs. A useful method for inducing the formation of human parasympathetic neurons has not yet been reported. Considering the antagonistic effects of noradrenergic and cholinergic inputs on target organs, it is important to generate both noradrenergic sympathetic and cholinergic parasympathetic neurons in the ANS for further application.

During NC development, parasympathetic neurons are considered to primarily be derived from cranial NC cells rather than from trunk NC cells from which only sympathetic neurons are derived. Recent studies have further demonstrated that parasympathetic neurons can arise from Schwann cell precursors, and this happens at a later stage than sympathetic neuron differentiation^13,14^. However, previous studies generating sympathetic neurons from hPSCs have not been able to generate cholinergic parasympathetic neurons in the ANS. These studies have induced trunk NC cells or sympathoadrenal cells to efficiently generate sympathetic neurons on the basis of the regulation of the dorso-ventral or anterior-posterior axis^10–12^ in hPSC differentiation pathways. We hypothesized that precise determination of trunk NC cells from hPSCs inhibit the induction of cholinergic parasympathetic neurons. Hence, a different approach for ANS specification of hPSCs is required to induce both sympathetic and parasympathetic ANS neurons.

Accordingly, herein, we attempted to develop a method to induce ANS progenitors capable of generating both sympathetic and parasympathetic neurons. Previous studies have generally used sonic hedgehog (SHH) agonist and retinoic acid (RA) to the modulate dorso-ventral and anterior-posterior axes for generating trunk NC and sympathoadrenal cells^11,12^. In contrast, we focused on signalling pathways from NC lineages to PNS subtypes. For example, the activation of WNT signalling promotes the specification of the sensory fate in cranial or trunk NC cells or sensory neuron precursors^15,16^, and the activation of SHH signalling promotes the specification of the enteric fate of vagal or sacral NC cells^17^. Furthermore, as generally used in the previous studies, bone morphogenetic protein (BMP) signalling activation is essential for autonomous specification of cranial or trunk NC cells^10–12^. Hence, we hypothesized that a stepwise induction strategy, including an NC induction step and subsequent autonomous specification step with restricted WNT signalling inhibition, SHH signalling inhibition, and BMP signalling activation can lead to the specification of hPSCs into ANS lineages, thus inducing noradrenergic sympathetic neurons and cholinergic parasympathetic neurons (Supplementary Fig. 1a). Through stepwise induction and selective culture conditions, we confirmed that hPSC-derived sympathetic-like and parasympathetic-like neurons regulated the beating rates of human induced pluripotent stem cell (hiPSC)-derived cardiomyocytes *in vitro*.

## Results

### *In vitro* derivation of neural crest cells and autonomic progenitors from hPSCs

**Figure 1:**
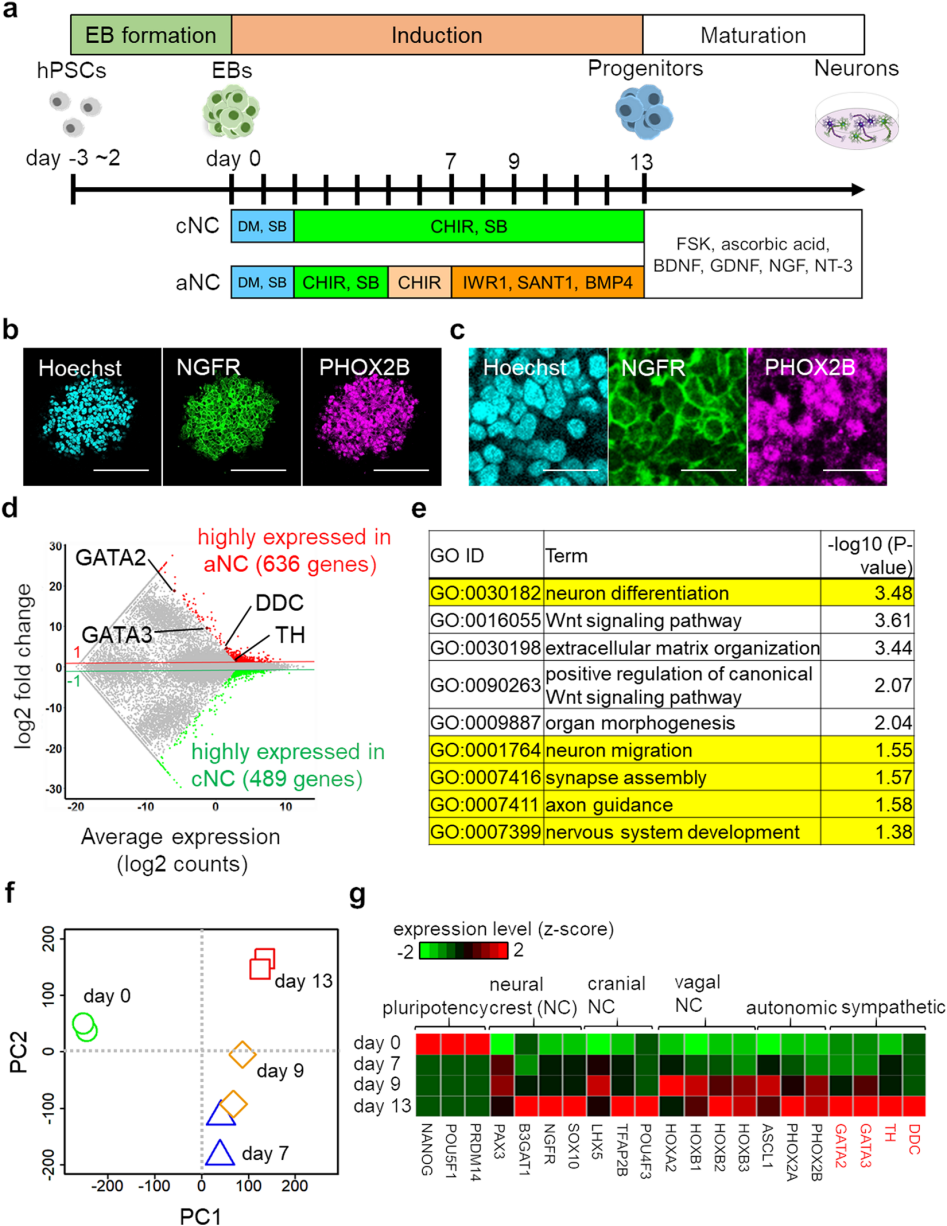
Stepwise treatment with small compound induces autonomous progenitor specification in pluripotent cells. **(a)** Schematic representation of induction methods. Compared to the conventional neural crest (cNC) method, we divided the period of induction of progenitors of the autonomic nervous system (ANS) into 4 steps during autonomic neural crest (aNC) induction. (**b)** Immunofluorescence staining of aNC-induced cells for NC markers NGFR and ANS marker PHOX2B on day 13. Scale bar; 100 μm. (**c**) Magnified immunofluorescent images of (**b**). Scale bar; 20 μm. (**d)** Global mRNA expression patterns between aNC-induced and cNC-induced cells on day 13. The MA plot shows differentially expressed genes (red genes, aNC enriched; green genes, cNC enriched) with sympathetic neuron marker genes. (**e)** Gene Ontology analysis of biological processes of genes enriched in aNC-induced cells on day 13. Neuronal differentiation- and development-related terms are highlighted in yellow. **(f)** Principal component analysis of RNA-seq data from aNC-induced cells on day 0, 7, 9, and 13 (*n* = 2 for each). (**g)** Selected developmental marker genes in RNA-seq data highlighting the differentiation of aNC-induced cells toward NC and ANS lineages. Sympathetic markers were identified and are highlighted in red.

We first aimed to develop a strategy to induce parasympathetic neurons in hPSC cultures. To this end, we modified a conventional NC (cNC) induction method for the optimal derivation of ANS lineages (aNC, Fig. 1a). WNT signalling activation and inhibition of both BMP and tumour growth factor (TGF)-β signalling are typically efficient and sufficient for NC induction^18,19^. Accordingly, in the aNC method, WNT activation via CHIR99021 (CHIR), BMP inhibition via dorsomorphin (DM), and TGF-β inhibition via SB431542 (SB) for NC induction were carried out for 7 days. This was followed by treatment with a combination of WNT signalling inhibitor (IWR1), SHH signalling inhibitor (SANT1), and recombinant human BMP4 for 6 days to promote the specification of cells into the ANS lineage, in contrast with treatment for 11 days with CHIR and SB in the cNC method. After 13 days of induction, immunofluorescence analysis revealed that over 90% of aNC-induced cells expressed NC marker genes NGFR and over 50% of cells expressed ANS-lineage marker PHOX2B (Fig. 1b,c), indicating that the aNC method induces hPSCs into NC cells, specifically including the ANS lineage. We then performed RNA-seq analysis and compared the difference in global mRNA expression in aNC- and cNC-induced cells on day 13 to validate our stepwise method for ANS induction. In the cNC method, WNT activation and TGF-β inhibition were maintained for 11 days, thus inducing general PNS neurons^18^. As summarized in the MA plot (Fig. 1d), 636 genes in aNC-induced cells and 489 genes in cNC-induced cells were significantly upregulated. Sympathetic markers GATA2, GATA3^20^, tyrosine hydroxylase (TH), and dopa decarboxylase (DDC), had were previously identified via the aNC method. Gene ontology (GO) analysis revealed that genes upregulated in aNC-induced cells were enriched in neuronal differentiation- and development-related GO terms (Fig. 1e). In contrast, genes upregulated in cNC-induced cells were enriched in translation- or cell cycle-related GO terms, indicating that these cells were immature (Supplementary Fig. 1b). Hence, the aNC method not only directs cells to the ANS linage but also enhances neuronal differentiation of hPSCs compared to the cNC method. To elucidate the differentiation mechanisms, we performed time-series RNA-seq analysis of aNC-induced cells. Principle component analysis and GO analysis revealed distinct and stepwise alterations during cell induction (Fig. 1f and Supplementary Fig. 1c–e). We then confirmed that NC-related genes (including PAX3, B3GAT1, NGFR, and SOX10), ANS lineage-related genes (including ASCL1, PHOX2A, and PHOX2B), and sympathetic neuron-related genes (including GATA2, GATA3, TH, and DDC) were gradually upregulated (Fig. 1g and Supplementary Fig. 1f). Remarkably, cranial NC marker genes (including LXH5, TFAP2B, and POU4F3^12,21^) and vagal NC marker genes (including HOXA2, HOXB1, HOXB2, and HOXB3) were upregulated. In addition, trunk NC related genes^22^ were also upregulated (Supplementary Fig. 1g), indicating the induced progenitors were heterogeneous cell populations. These results indicate that our stepwise method induce the differentiation of hPSCs into ANS progenitors via NC lineage stage.

### Differentiation of functional sympathetic-like and parasympathetic-like neurons from hPSC-Derived autonomic progenitors

**Figure 2:**
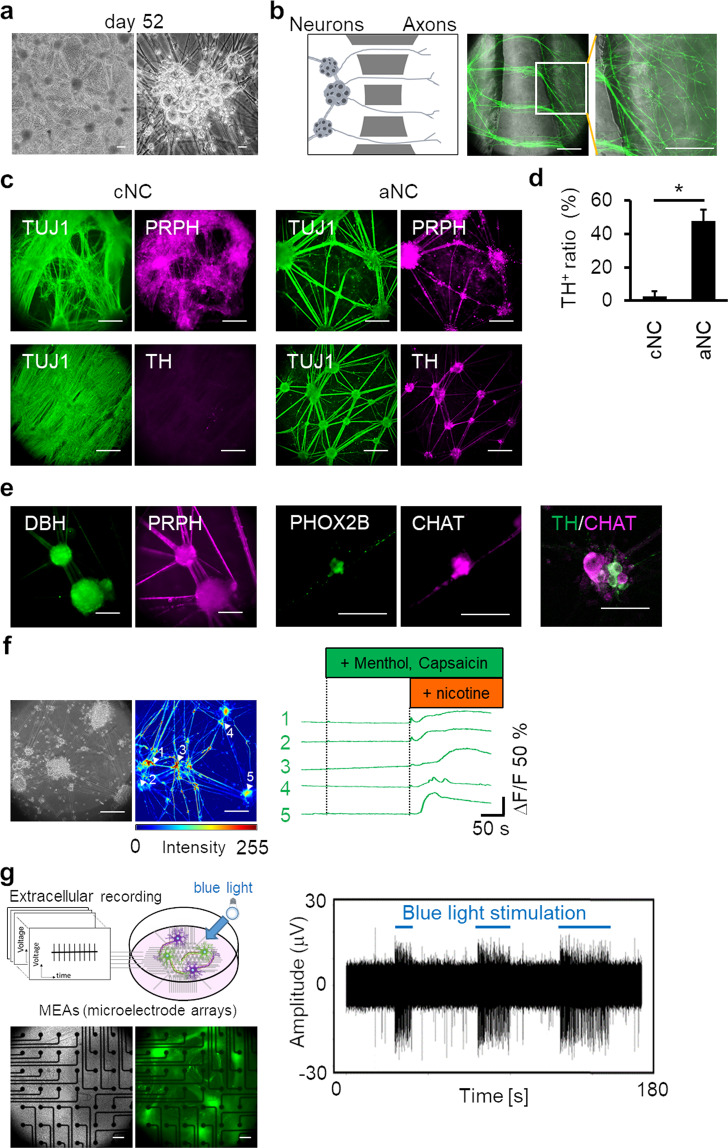
Human pluripotent stem cell (hPSC)-derived progenitors of the autonomic nervous system (ANS) differentiate into functional ANS neurons. **(a**) Phase-contrast images of differentiated neurons on day 52. We induced hPSC-derived autonomic progenitors to mature neurons, using the autonomic neural crest (aNC) method shown in Fig. 1a. Scale bar; left 100 μm, right 10 μm. (**b**) Distinct axon elongation of differentiated neurons on day 36. Neurons were cultured in the microfabricated device and labelled with a Synapsin-1-GFP lentiviral vector to detect axons. Neurons plated on the left-side chamber extended axons to the right-side chamber via microtunnels of 50-μm width (left panel). A highly magnified image of the boxed region in the middle panel is shown (right). (**c**) Immunofluorescence staining for TUJ1, PRPH, and TH on day 36. (**d)** Comparison of the ratio of TH-positive to TUJ1-positive cells (*n* = 5, error bar shows SDs; two-sided Student’s unpaired *t*-test *P < 0.001). The ratio of TH-positive areas to TUJ1-positive areas in induced neurons was calculated. (**e)** Immunofluorescence staining of DBH and PRPH for sympathetic-like neurons on day 68 and PHOX2B and CHAT for parasympathetic-like neurons on day 52. Double immunofluorescence staining for TH and CHAT on day 59 were also shown. (**f**) Typical traces of calcium transients of induced neurons with indicated drugs. We used sensory neuron agonists menthol and capsaicin and ANS agonist nicotine. The right panel shows representative traces of calcium transients in 5 cells as shown in the phase-contrast image (left panel) for 350 s (see also Supplementary Movie 2). The colour bar shows the fluorescent intensity. (**g**) Representative electrical signal in a selected single electrode after illumination. ChR2-expressing neurons were cultured on MEA substrates, and electrical activity was recorded. The left panel shows a schematic representation of the experimental protocol. Middle panels show phase-contrast and fluorescence microscopic images of neurons plated on MEA substrate. Blue lines indicate the timing of blue light stimulation in the right panel. Scale bar; 100 μm in (**b,c,e–g**).

After aNC induction, we guided hPSC-derived ANS progenitors into mature neurons (Fig. 1a). Induced ANS progenitors were collected, enzymatically dissociated, and plated onto conventional culture plates. For maturation, we continued the neuronal cultures for at least 3 weeks. Interestingly, the cells spontaneously formed ganglion-like structures in samples on day 52, and these ganglion-like structures were interconnected via neurite bundles (Fig. 2a). We then examined the capability of axon elongation in induced cells. To isolate axons, day 13 ANS progenitors were plated onto the microfabricated device comprising microtunnels, facilitated the passage of only axons^23,24^. On day 36, we confirmed that axons of Synapsin-1-GFP-labelled cells passed through the microtunnels (Fig. 2b). These results indicate that neurons were generated from the induced ANS progenitors (see also Supplementary Movie 1). Forty-eight percent of peripheral neurons (TUJ1^+^/PRPH^+^) were TH^+^ upon aNC induction, indicating the induction of sympathetic-like neurons. However, only 2% of peripheral neurons were TH^+^ upon cNC induction (Fig. 2c,d). Further, sympathetic-like and parasympathetic-like neurons, marked with TH or dopamine b-hydroxylase (DBH)/PRPH, PHOX2B/choline acetyltransferase (CHAT), were separately generated (Fig. 2e). The outcomes of the aNC methods were consistent when we used other hPSC cell lines including H1 human embryonic stem cells (hESCs) or 253G1 hiPSCs (Supplementary Fig. 2a,b), highlighting the versatility of our method. Moreover, we confirmed the generation of chromaffin cells by analysing the chromaffin cell marker expression of phenylethanolamine *N*-methyltransferase (PNMT), as these cells share common progenitors with sympathetic or parasympathetic neurons^25,26^ (Supplementary Fig. 2c). Furthermore, sympathetic-like neurons were more rapidly induced (day 13) than parasympathetic-like neurons (day 21) (Supplementary Fig. 2d). This order of neuronal induction was consistent with that of murine ANS^13,14^. Hence, aNC-induced cells first generate sympathetic-like neurons, followed by parasympathetic-like neurons.

Calcium imaging and electrophysiological analysis were performed to explore the functions of generated neurons. First, we confirmed that the generated neurons had peripheral properties including rare spontaneous activity and reproducible responses to electrical stimulation (Supplementary Fig. 2e–g). These results were consistent with previously reported characteristic of PNS neurons^23,27^. Second, we analysed calcium signal responses to nicotine, which activates nicotinic acetylcholine receptors (nAChRs) of post-ganglionic neurons in the ANS. As expected, these neurons strongly responded to nicotine application at day 49 sample, whereas they rarely responded to the TRPM8 agonist menthol and the TRPV1 agonist capsaicin, which classically activate sensory neurons (Fig. 2f, Supplementary Movie 2). By contrast, cNC-induced neurons responded to menthol and capsaicin, not to nicotine (Supplementary Fig. 2h, Supplementary Movie 3). This supported that cNC method majorly induced sensory neurons. Furthermore, we established an optogenetic system (channelrhodpsin-2 (ChR2) expression in the neuron) and plated neurons onto microelectrode arrays (MEAs), and then confirmed blue-light evoked activity of induced neurons (Fig. 2g). These results indicate that a stepwise method can induce functional ANS neurons.

### Characterization of differentiated sympathetic-like and parasympathetic-like neurons by single-cell RNA-Seq profiling

**Figure 3:**
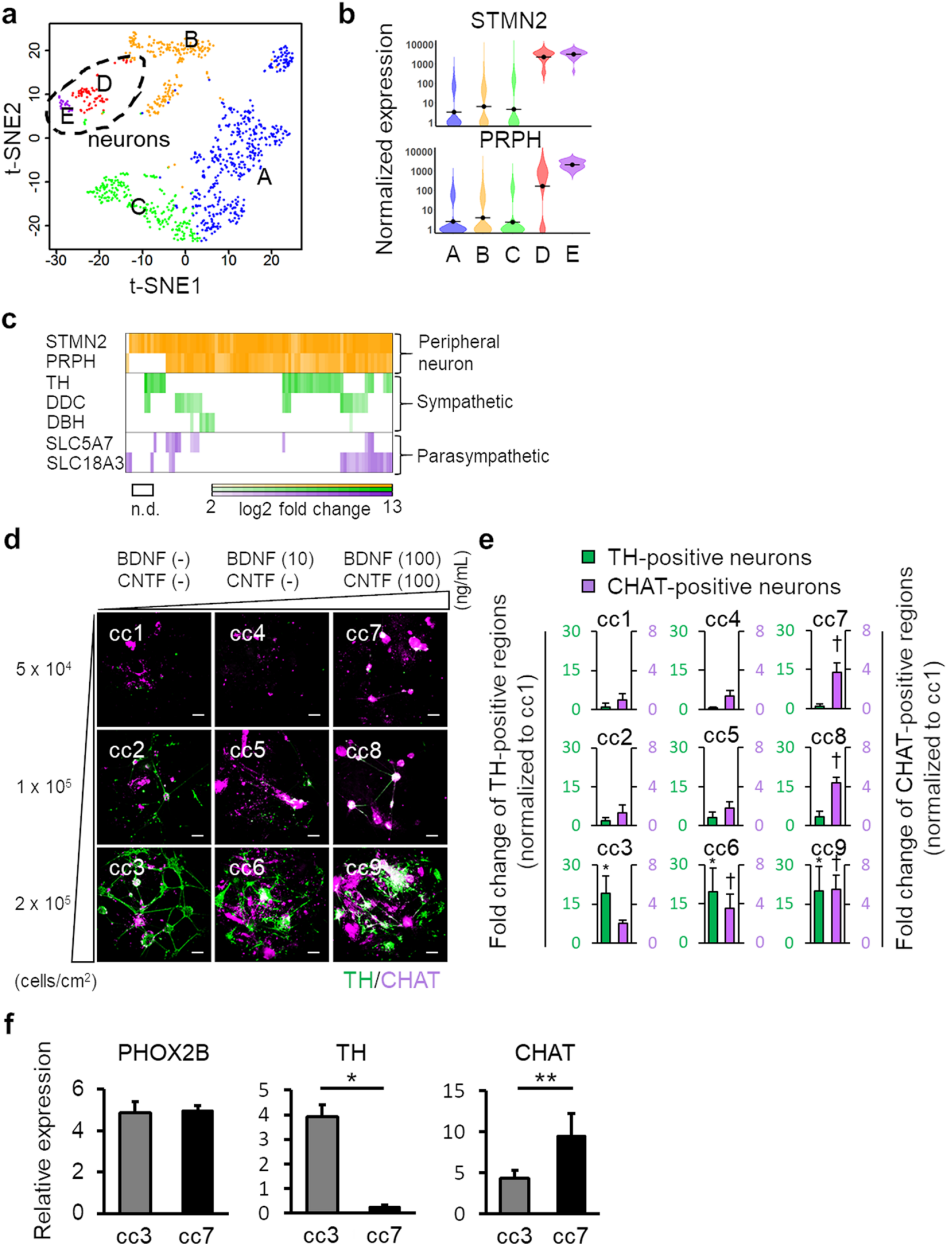
Cell density and neurotrophic factor facilitate selective differentiation of sympathetic-like and parasympathetic-like neurons. (**a**) Single-cell RNA-seq analysis using differentiated cells on day 58. The t-distributed stochastic neighbor embedding (t-SNE) visualization showing 5 distinct cell populations (clusters A–E) based on K-means clustering. (**b**) Violin plots indicating the expression levels of STMN2 and PRPH across five clusters. (**c**) Expression patterns of peripheral neuron markers, sympathetic neuron markers, and parasympathetic neuron markers in each neuron of cluster D and E. (**d**) Immunofluorescence analysis of 9 culture conditions. Scale bar; 100 μm. (**e**) Expression fold-changes in TH-positive and CHAT-positive neurons under 9 culture conditions. Each result is normalized to the positive region in the cc1 condition (*n* = 3; error bar shows SDs; one-way ANOVA Tukey post-test *P < 0.05 compared to cc1 for TH-positive neurons. one-way ANOVA Tukey post-test ^†^*P* < 0.05 compared to r1 condition for CHAT-positive neurons). (**f**) mRNA expression levels of PHOX2B, TH, and CHAT under each culture condition (*n* = 3; error bar shows SDs; two-sided Student’s unpaired *t*-test for PHOX2B and CHAT. Welch’s *t*-test for TH. *P < 0.01, **P < 0.05).

To clarify the identities of hPSC-derived sympathetic-like and parasympathetic-like neurons, we performed single-cell RNA-seq analysis of 993 cells on day 58. We initially performed 2D visualization of RNA-seq data through t-distributed stochastic neighbor embedding (t-SNE) with k-means clustering (*k* = 5) to categorize the data into distinct clusters (Fig. 3a). Based on STMN2 and PRPH (PNS neuronal markers) expression levels, clusters D and E represented neuronal populations (Fig. 3b). GO analysis of genes enriched in each cluster also indicated that clusters D and E comprised neurons (Supplementary Fig. 3a and Supplementary list). Considering the enriched genes, neurons in cluster D would be almost sympathetic neurons, and that cells in cluster E would be heterogenetic populations which include enteric, sympathetic, and parasympathetic neurons. In addition, stromal cells would be included in other clusters, particularly in cluster B. To assess differences between non-neuronal and neuronal populations, we re-categorized all cells into two populations for global transcriptome analysis (Supplementary Fig. 3b). We identified 1702 significantly upregulated genes, including sympathetic and parasympathetic markers, and various neuronal-associated genes (Supplementary Fig. 3,b,c). We then determined the number of sympathetic-like neurons expressing at least one sympathetic marker (TH, DDC, or DBH), and parasympathetic-like neurons expressing at least one parasympathetic marker (SLC5A7 or SLC18A3). We identified 31 sympathetic-like neurons and 8 parasympathetic-like neurons among 87 neurons (Fig. 3c). However, 22 neurons expressed both sympathetic and parasympathetic markers. Hence, the proposed differentiation method did not effectively distinguish differentiation pathways toward sympathetic and parasympathetic neurons.

### Cell density and neurotrophic factors are key parameters in the specification of sympathetic-like and parasympathetic-like neurons

To overcome the differentiation uncertainty of ANS neurons, we optimized the neuronal differentiation method to precisely derive sympathetic and parasympathetic neurons. In cultured sympathetic neurons, the cell density and cell-cell contacts differentially regulate transmitter phenotype expression^28^. Thus, we predicted that cell density changes influence sympathetic or parasympathetic determination. In contrast, neurotrophic factors, particularly brain-derived neurotrophic factor (BDNF) and ciliary neurotrophic factor (CNTF) influence the properties of sympathetic neurons in neonatal rats^29^. Together, we modulated differentiation pathways per these two parameters. We evaluated 9 culture conditions (cc1–9) and calculated the generation ratios of sympathetic-like and parasympathetic-like neurons from TH and CHAT expression (Fig. 3d,e). As expected, cell density influenced sympathetic neuronal differentiation. In contrast, BDNF and CNTF levels are key factors regulating parasympathetic neuronal differentiation. Consequently, we defined the cc3 condition (high cell density, without BDNF and CNTF) as a sympathetic-differentiation method and the cc7 condition (low cell density, high levels of BDNF and CNTF) as a parasympathetic neuron differentiation method. qPCR analysis revealed biased TH or CHAT expression in neurons subjected to the sympathetic- and parasympathetic differentiation methods, whereas both types of neurons expressed similar levels of PHOX2B (Fig. 3f). We also examined whether sympathetic-like neurons were destroyed by 6-hydroxydopamine (6-OHDA), a specific catecholaminergic neurotoxin (Supplementary Movie 4), whereas 6-OHDA-treated parasympathetic-like neurons under the same conditions remained unaffected (Supplementary Movie 5). These results indicate highly selective induction of sympathetic-like and parasympathetic-like neurons from hPSCs.

### Stimulation of sympathetic-like and parasympathetic-like neurons leads to control of spontaneous beating of hiPSC-derived cardiomyocytes in an opposing manner

**Figure 4:**
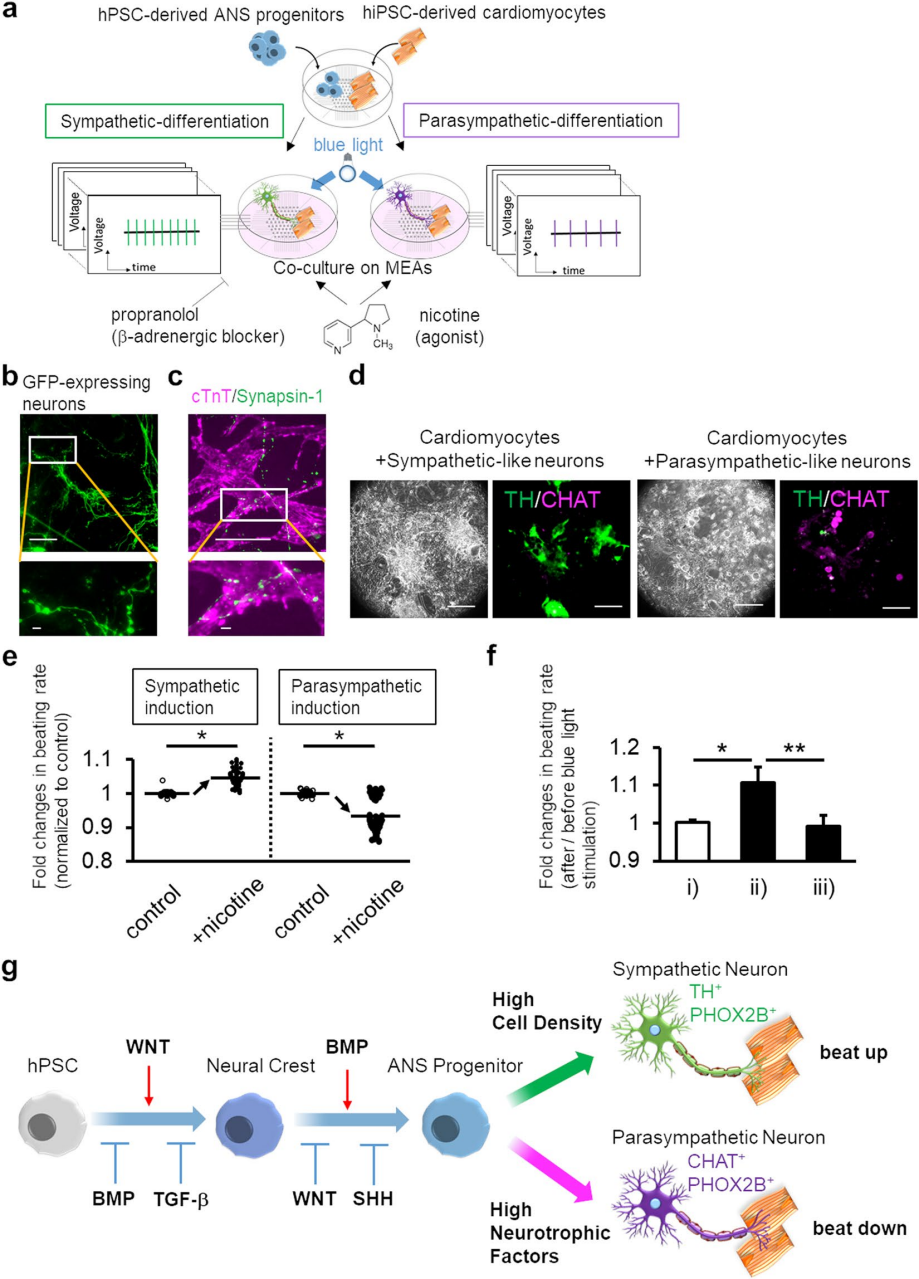
Human pluripotent stem cell (hPSC)-derived sympathetic-like and parasympathetic-like neurons regulate the beating of human induced PSC (hiPSC)-derived cardiomyocytes through antagonistic effects. **(a**) Schematic representation of the generation of hPSC-derived sympathetic-like or parasympathetic-like neurons and cardiomyocytes. Nicotine or blue-light illumination were applied to activate neurons and cardiac signals were measured using MEA. β-Blocker (propranolol) was also applied to co-cultured samples with sympathetic-like neurons and cardiomyocytes. (**b**) Fluorescence image of GFP expression which induced neurons showing varicosity-like structures in neurite regions under co-culture conditions. Highly magnified image of the boxed region in the top image is shown (bottom). Scale bar; top 100 μm, bottom 10 μm. (**c**) Immunofluorescence staining for cTnT among cardiomyocytes and Synapsin-1. The magnified image of the boxed region in the top image is shown (bottom). Scale bar; top 100 μm, bottom 10 μm. (**d**) Immunofluorescence staining for TH and CHAT in co-cultures with cardiomyocytes. Scale bar; 100 μm. (**e**) Fold-changes in the rate of cardiomyocytes beating upon nicotine stimulation (20 μM). Each beating rate was calculated from 30 local field potentials before and after nicotine application (see also Supplementary Fig. 4) (*n* = 90 from 3 samples, two-sided Student’s unpaired *t*-test *P < 0.00001). (**f**) Fold-changes in the rate of cardiomyocyte beating with photoactivation of ChR2-expressing sympathetic-like neurons. Measurements were carried out under three conditions: (i) photoactivation of cardiomyocytes, (ii) photoactivation of co-cultured ChR2-expressing sympathetic-like neurons and cardiomyocytes, and (iii) photoactivation of co-cultured ChR2-expressing sympathetic-like neurons and cardiomyocytes in the presence of 1 μM propranolol (*n* = 3; error bar shows SDs; one-way ANOVA Tukey post-test *P < 0.01, **P < 0.05). (**g**) A schematic representation of derivations of human sympathetic-like and parasympathetic-like neurons from pluripotent stem cells. Restricted WNT activation and inhibition with modulation of SHH and BMP signalling induced the specification of ANS progenitors. Along with stepwise induction, controlled culture conditions led to precise generation of ANS neurons, facilitating the regeneration of cardiac ANS *in vitro*.

To examine the precise regulation of tissue functions *in vitro* via sympathetic and parasympathetic signals, we co-cultured these neurons with human iPSC-derived cardiomyocytes. ANS progenitors on day 13 and cardiomyocytes were plated onto MEAs, and ANS progenitors were differentiated into sympathetic-like (cc3 condition) or parasympathetic-like neurons (cc7 condition) through selective differentiation methods (Fig. 4a). During co-culture, the neurons formed varicosity-like swollen structures along their neurites, similar to endogenous ANS neurons (Fig. 4b), positive for Synapsin-1 and overlapped with cTnT-positive cardiomyocytes (Fig. 4c). Varicosities are important for ANS innervations because varicosities contain neurotransmitters released at numerous and distinct locations at target cells. Hence, the generated neurons physically interacted with cardiomyocytes. We confirmed that these neurons were successfully differentiated upon co-culturing (Fig. 4d). After 3 weeks of co-culture, we examined the effects of neuron stimulation on cardiomyocyte activity. On stimulating sympathetic-like neurons with nicotine, the beating rates significantly increased; however, on stimulating parasympathetic-like neurons, the beating rates significantly decreased (Fig. 4e and Supplementary Fig. 4a). Notably, nicotine did not affect cardiomyocyte beating without neurons (Supplementary Fig. 4b). In addition, we confirmed that spontaneous beating rates of co-cultured cardiomyocytes distinctly increased compared to cardiomyocytes without neurons (Supplementary Fig. 4b), indicating maturation of cardiomyocytes^10^. To further examine the regulation of the beating rate by the induced neurons, we stimulated ChR2-expressing sympathetic-like neurons with blue light. As expected, the beating rates significantly increased upon light stimulation, and this optogenetic effect was inhibited in the presence of the β-signal blocker propranolol, indicating that changes in beating were caused by β-adrenergic associations (Fig. 4f, Supplementary Movie 6). Hence, the generated neurons coupled with human cardiomyocytes to regulate their beating rate in a neuronal property-dependent manner. The overall study methodology and findings are summarized in Fig. 4g.

## Discussion

In this study, we provide a highly selective induction method for sympathetic-like and parasympathetic-like neurons from hPSCs. Taken together with recent studies focusing on deriving sympathetic neurons from hPSCs^10–12^, our techniques provide insights into *in vitro* modelling of human ANS innervations. Acquisition of ANS functions highlights the simplicity of our method of inducing human sympathetic and parasympathetic neurons and reconstructing tissues that can be stimulated by both neurons.

Our selective differentiation methods potentially facilitate modelling and investigation of human ANS innervations, such as regulating pancreatic insulin secretion under healthy and diabetic conditions. Although we confirmed neuronal regulation in functional co-culture assays, we did not detect noradrenaline or acetylcholine release in culture media upon ELISA (data not shown), probably because of rapid degradation by synaptic enzymes and their low, unquantifiable levels. Further high-sensitive detection methods including expansion culture are required to assess and quantify neurotransmitter release. Furthermore, photoactivation of ChR2-expressing parasympathetic-like neurons did not particularly influence the beating rates of co-cultured cardiomyocytes (data not shown), probably because the number of ChR2-expressing parasympathetic-like neurons was relatively small. Further optimization of an optogenetic system for parasympathetic neurons is required for high-grade ANS innervation modelling.

Neurotropic factors regulate differentiation pathways *in vivo* and *in vitro*^30–32^. Accordingly, we controlled the direction of ANS differentiation. Furthermore, cell density contributed to regulated ANS differentiation^28^. Since tissues and cells react to physical/mechanical stress *in vivo* and *in vitro*^33,34^, cell density might regulate cell fate through mechanical stress-based signalling. The biophysics of sympathetic/parasympathetic differentiation with mechanical signalling, including the YAP/TAZ-TEAD pathway, is worth investigating.

Single-cell analysis identified neurons expressing sympathetic and parasympathetic markers (Fig. 3c), thus leading to the speculation of whether parasympathetic marker-positive neurons herein are actually parasympathetic neurons differentiated directly from ANS progenitors or cholinergic sympathetic neurons converted from noradrenergic sympathetic neurons herein. During early development, parasympathetic neurons transiently co-express sympathetic markers including TH and DBH^35,36^, and sympathetic neurons co-express parasympathetic markers^37,38^, indicating the possibility that double-positive neurons are immature or intermediate neurons. However, as described above, traditional *in vitro* studies and recent *in vivo* studies reported that sympathetic neurons acquire cholinergic properties via the effects of trophic factors including BDNF, CNTF^29,32,39,40^, and leukaemia inhibitory factor^41,42^. Furthermore, HMX2 and HMX3 were not upregulated or relevant herein, being newly identified definitive parasympathetic markers in mice^43^, in the induced ANS progenitors and induced neurons (data not shown). Although it remains unclear whether these new findings regarding parasympathetic neurons in mice are applicable among humans, the present results suggest that the parasympathetic-like neurons herein are not parasympathetic. On the other hand, chromaffin cells are also derived from Schwann cell progenitors, which also give rise to parasympathetic neurons^13,14^. Although we have not confirmed the involvement of Schwann cell precursors in the induction of parasympathetic-like neurons or chromaffin cells, Therefore, we considered that the generation of PNMT-positive chromaffin cells (Supplementary Fig. 2c) did not completely eliminate the possibility of the induction of parasympathetic neurons. Further studies are required to identify the origins or developmental pathways of human parasympathetic neurons *in vitro*, such as whether the intermediate Schwann cell precursor stage is involved.

In summary, we propose a novel *in vitro* co-culture system combining human ANS neurons and target tissues, thus facilitating *in vitro* analysis of tissue homeostasis, development, or pathological relevance of ANS innervations. We hence developed a selective differentiation method for hPSC-derived sympathetic-like and parasympathetic-like neurons, which exerted antagonistic effects on human cardiomyocyte dynamics *in vitro*. This study provides fundamental experimental platforms for basic and clinical research on neuronal-related cardiac diseases including arrhythmia or sudden death and for drug discovery for ANS-related disorders.

## Methods

### Cell lines

hiPSC lines 201B7 (female) and 253G1 (female) were obtained from RIKEN BRC (Tsukuba, Japan). hESC line H1 (male) was obtained from Wicell Research Institute (Madison, WI, USA). hiPSCs and hESCs were cultured in mTeSR1 WO 2ME/MV medium (Stemcell Technologies, Vancouver, BC, Canada).

iCell cardiomyocytes (hiPSC-derived cardiomyocytes; female) were obtained from FUJIFILM Cellular Dynamics (Madison, WI, USA) and cultured in accordance with the manufacturer’s instructions.

### Cell culture

The human iPS (201B7 and 253G1 line) and ES (H1 line) cells were maintained in mTeSR1 WO 2ME/MV medium (Stemcell Technologies) on Laminin511-E8 (iMatrix511; Nippi, Tokyo, Japan)-coated culture plates at 37 °C in a 5% CO_2_ incubator. The culture medium was changed daily. When the cells approached confluence, the colonies were digested into single cells using Accutase (Thermo Fisher Scientific, Waltham, MA, USA) and the resulting cells were sub-cultured or induced as described below.

Before initiating induction, the cells were transferred into 6-well plates (Corning, Inc., Corning, NY, USA) coated with 2-methacryloyloxyethyl phosphorylcholine (MPC) (Lipidure CM5206E; NOF, Tokyo, Japan) at a density of 1–2 × 10^5^ cells/cm^2^. The MPC-coated culture plates were incubated on a rotary shaker at 95 rpm (OS-762RC, Optime, Tokyo, Japan). They were maintained in mTeSR1 WO 2ME/MV medium containing 10 μM Y-27632 (FUJIFILM Wako Pure Chemical Industries, Osaka, Japan) for 2 or 3 d to form embryonic bodies (EBs). Induction was initiated as follows. EBs were transferred into and cultured in knockout serum replacement (KSR) medium containing 2 μM dorsomorphin (DM; Sigma-Aldrich, St. Louis, MO, USA), 10 μM SB431542 (SB; Sigma-Aldrich), and 10 ng/mL bFGF (FUJIFILM Wako Pure Chemical Industries) for 2 d (day 0–2). KSR medium comprised DMEM-F12 (FUJIFILM Wako Pure Chemical Industries), 20% KSR (Life Technologies, Carlsbad, CA, USA), 1% non-essential amino acids (FUJIFILM Wako Pure Chemical Industries), 1% monothioglycerol (FUJIFILM Wako Pure Chemical Industries), and 1% penicillin-streptomycin (FUJIFILM Wako Pure Chemical Industries). During days 2–5, the EBs were transferred into and cultured in KSR medium containing 3 μM CHIR99021 (CHIR; Cayman Chemical, Ann Arbor, MI, USA), 20 μM SB, and 10 ng/mL bFGF. On days 5–7, EBs were transferred to and cultured in a mixture of KSR/N2 medium (3:1) containing 3 μM CHIR99021 and 10 ng/mL bFGF. The N2 medium comprised DMEM-F12, 1% N2 supplement (FUJIFILM Wako Pure Chemical Industries), 1% non-essential amino acids, and 1% penicillin-streptomycin. On days 7–9, the EBs were transferred to and cultured in a mixture of KSR/N2 medium (1:1) containing 10 μM IWR1 (Sigma-Aldrich), 250 nM SANT1 (Sigma-Aldrich), 25 ng/mL recombinant human bone morphogenetic protein 4 (BMP4; FUJIFILM Wako Pure Chemical Industries), and 10 ng/mL bFGF. During days 9–13 (medium change at day 12), the EBs were transferred to and cultured in a mixture of KSR/N2 medium (1:3) containing 10 μM IWR1 (Sigma-Aldrich), 250 nM SANT1 (Sigma-Aldrich), 25 ng/mL BMP4, and 10 ng/mL bFGF. At day 13, induced EBs were dissociated with TrypLE Express (Thermo Fisher Scientific) and the cells were plated on poly-l-ornithine (Sigma-Aldrich)/laminin (Sigma-Aldrich)-coated culture plates in neuronal differentiation (ND) medium comprising N2 medium, 10 μM forskolin, 50 μg/mL ascorbic acid, 10 ng/mL recombinant human brain-derived neurotrophic factor (BDNF), 10 ng/mL recombinant human glial-cell-derived neurotrophic factor, 10 ng/mL recombinant human nerve growth factor-β, and 10 ng/mL recombinant human neurotrophin 3 (all from FUJIFILM Wako Pure Chemical Industries). The cells were plated at densities of 5 × 10^4^ to 2 × 10^5^ cells/cm^2^. The ND medium was changed twice per week.

For selective differentiation experiments on sympathetic and parasympathetic neurons, recombinant human ciliary neurotrophic factor (CNTF; FUJIFILM Wako Pure Chemical Industries) was added to the ND medium. The concentrations of BDNF and CNTF were changed under each culture condition, ranging from 0 to 100 ng/mL.

To generate peripheral neurons for the control method (cNC method, see Fig. 1), the same induction method was followed until day 5. During days 5–13 (medium change every 2 days), EBs were repeatedly cultured in KSR medium containing 3 μM CHIR, 20 μM SB, and 10 ng/mL bFGF. On day 13 and thereafter, neuronal maturation was carried out as described above.

To culture induced neurons in the microfabricated device, we used soft lithography and replica moulding techniques as previously reported^23^. Briefly, SU-8 3005 (Microchem Corp., Newton, MA, USA) was used to produce the master mould for microtunnels with height of 5 μm and width of 50 μm. A mixture of polydimethylsiloxane-prepolymer and curing catalyst (10:1 weight ratio; Silpot 184; Dow Corning Corp., Midland, MI, USA) was poured over the moulds and cured at 70 °C for 1 h. Thereafter, the polydimethylsiloxane sheet was released from the moulds and attached to a cell culture dish.

Human PSC experiments were approved by the Ethics Committee of the National Institute of Advanced Industrial Science and Technology. The use of human ES cells was approved by the Ministry of Education, Culture, Sports, Science, and Technology (MEXT) of Japan. Furthermore, the use of human ES cells was carried out in accordance with “Guidelines on the Utilization of Human Embryonic Stem Cells” of MEXT of Japan.

### Immunochemical staining

Immunochemical experiments were performed as previously reported^23^. Briefly, the samples were fixed with 4% paraformaldehyde (FUJIFILM Wako Pure Chemical Industries) in phosphate-buffered saline (PBS; Thermo Fisher Scientific) for 20 min, permeabilized with 0.1% Triton X-100 (FUJIFILM Wako Pure Chemical Industries) in PBS for 10 min, and blocked with PBS containing 4% Block Ace (DS Pharma Biomedical, Osaka, Japan) and 0.1% Triton X-100 for 1 h at room temperature. The following primary antibodies were used: mouse anti-NGFR (1:200; Advanced Targeting Systems, San Diego, CA, USA), mouse anti-class III beta-tubulin (TUJ1; 1:1000; Abcam, Cambridge, UK), mouse anti-microtubule-associated protein (MAP2; 1:1000; Abcam), mouse anti-PHOX2A (1:50; Abcam), mouse anti-PHOX2B (1:100; Proteintech, Rosemont, IL, USA), mouse anti-tyrosine hydroxylase (TH; 1:200; Merck Millipore, Billerica, MA, USA), mouse anti-dopamine β-hydroxylase (DBH; 1:50; Santa Cruz Biotechnology, Dallas, TX, USA), mouse anti-phenylethanolamine *N*-methyltransferase (PNMT; 1:100; Abcam), rabbit anti-SOX10 (1:500; Abcam), rabbit anti-Peripherin (PRPH; 1:1000; Merck Millipore), rabbit anti-TH (1:500; Merck Millipore), rabbit anti-PHOX2B (1:100; Abcam), and rabbit anti-choline acetyltransferase (CHAT; 1:1000; Abcam).

### Calcium imaging

Samples were incubated with 5 mg/mL fluo-4 AM, a calcium indicator (Thermo Fisher Scientific), in NM medium at 37 °C for 30 min. Thereafter, NM medium containing fluo-4 AM was replaced with Ringer’s solution (148 mM NaCl, 2.8 mM KCl, 2 mM CaCl_2_, 1 mM MgCl_2_, 10 mM HEPES, and 10 mM glucose; pH 7.4). The sample was placed on the stage of an inverted microscope (IX81; Olympus, Tokyo, Japan), and fluorescence was detected with an electron multiplying charge-coupled device camera (iXon; Andor, Belfast, UK). For electrical stimulation, a pair of platinum electrodes (0.5 mm diameter; Nilaco) was immersed in the solution, and electrical pulses with a constant voltage were delivered to the cells at 10-s intervals using an electrical stimulator (SEN-3401; Nihon Kohden, Tokyo, Japan). Negative phase pulses with durations of 3 ms and an intensity of 10 V were used. For drug application, 10 μM capsaicin (in ethanol; Sigma-Aldrich), 100 μM menthol (in ethanol; Sigma-Aldrich), and 40 μM nicotine (in ethanol; Sigma-Aldrich) were added to Ringer’s solution during fluorescent observation. A frame rate of 2/s was used. The recorded fluorescent signals were analysed using ImageJ software^44^ (National Institutes of Health, Bethesda, MD, USA; available at http://imagej.nih.gov/ij/).

### RNA-Seq

Total RNA was isolated from the ReliaPrep RNA Cell Miniprep System (Promega, Madison, WI, USA). The purity and concentration of RNA were determined using a NanoDrop Lite spectrophotometer (Thermo Fisher Scientific). The following RNA-seq operations were conducted at Macrogen (Seoul, Korea). The library was constructed using more than 1 μg of cDNA and qualitatively assessed using the Agilent 2100 Bioanalyzer (Agilent Technologies). The resulting library was sequenced with a NovaSeq. 6000 (Illumina, San Diego, CA, USA) to produce 150-bp paired-end reads. Using hisat2 (ver. 2.1.0), the obtained reads were mapped to the human hg38 genome with trimming 10 bases from the 5′ end and 80 bases from the 3′ end. The mapped reads were converted to fragments per kilobase of exon per million mapped sequence reads (FPKM), using cufflinks (ver. 2.2.1) and gene annotation data provided by Illumina iGenome. The FPKM values were quantile normalized using an in-house R script for subsequent analysis. We selected transcripts with FPKM values of >1 for subsequent analysis to exclude downregulated transcripts from the analysis. We defined a gene as differentially expressed if its FPKM fold-change was more than 2 between conditions. The raw sequences in FASTQ format are available on the DNA data bank of Japan (DDBJ) (DRA008963).

### Single-cell RNA-seq

Single-cell RNA-seq was conducted at the Center for Omics and Bioinformatics, University of Tokyo, Japan. Briefly, day 58 induced ANS neurons were dissociated with TrypLE Express. The single-cell RNA-seq library was constructed using the Chromium Controller and Chromium Single Cell 3′ Reagent Kits v2 (10x Genomics, Pleasanton, CA, USA) with 1 × 10^5^ live cells. The library was sequenced using HiSeq. 3000 (Illumina) in accordance with the manufacturer’s instructions to obtain 100-bp paired-end reads. After sequencing, FASTQ files were generated using Cell Ranger ver. 2.1.0 mkfastq (10x Genomics). The obtained FASTQ files were mapped to the reference human genome GRCh38 obtained from 10x Genomics, and the mapped reads were enumerated using the Cell ranger count pipeline with default parameters. Dimensionality reduction with the tSNE algorithm for visualization and k-means clustering was performed using Cell Ranger. For volcano plot analysis, we set a significant threshold of adjusted *P*-value < 0.05 and Universal Molecular Indices (UMI) count-fold change >16. The raw sequences in FASTQ format are available on DDBJ (DRA009071).

### 6-Hydroxydopamine (6-OHDA) treatment

6-OHDA (Sigma-Aldrich) in PBS was freshly prepared before each experiment. The samples were treated with 100 μM 6-OHDA for 1 h and then replaced with fresh culture medium. To visualize the neurons, the samples were labelled with a lentiviral vector constructed to drive GFP cDNA using the human Synapsin-1 promoter (Synapsin-1-GFP). Time-lapse imaging of the 6-OHDA-treated samples were performed with JuLi FL (NanoEntek, Seoul, Korea) at every 5 min during imaging.

### qRT-PCR analysis

Total RNA was isolated from both selected and non-selected cells, using the ReliaPrep RNA Cell Miniprep System (Promega) in accordance with the manufacturer’s instructions. The purity and concentration of RNA were determined using a NanoDrop Lite spectrophotometer (Thermo Fisher Scientific). One hundred nanograms of total RNA were reverse-transcribed to cDNA using the ReverTra Ace qPCR RT Kit (TOYOBO, Osaka, Japan). qRT-PCR was then performed using the PikoReal 96 Real-Time PCR system (Thermo Fisher Scientific) with THUNDERBIRD SYBR qPCR Mix (TOYOBO). The expression values were normalized to that of *36b4* and are reported as mean ± standard deviation (SD) values of triplicate measurements. The following primers were used: CHAT, forward 5′-GCCTTCTACAGGCTCCATCG-3′, reverse 5′-GGAGTGGCCGATCTGATGTT-3′, PHOX2B, forward 5′-GCTGGCCCTGAAGATCGAC-3′, reverse 5′-TCAGACTTTTTGCCCGAGGAG-3′, TH, forward 5′-GCGCAGGAAGCTGATTGC-3′, reverse 5′-CAATCTCCTCGGCGGTGTAC-3′, 36B4, forward 5′-AGATGCAGCAGATCCGCA-3′, reverse 5′-GTTCTTGCCCATCAGCACC-3′.

### Viral infection

To visualize induced neurons from hPSCs, a lentiviral vector harboring green fluorescent protein (GFP) cDNA and neomycin resistance genes and the human Synapsin-1 promoter (Synapsin-1-GFP)^45^ was used. The cells were infected with the Synapsin-1-GFP vector. After 24 h, cells were transferred to fresh culture medium without the vector. Time-lapse neuronal imaging was performed using an IX81 inverted microscope and Metamorph at every 30 min during imaging.

### Microelectrode array (MEA) recording of cardiomyocyte dynamics

Human iPS-derived cardiomyocytes (iCell cardiomyocytes; FUJIFILM Cellular Dynamics, Madison, WI, USA) were plated and cultured onto MEA substrates with embedded 64 electrodes (M64-GL1-30Pt; Axion BioSystems, Inc., Atlanta, USA). Extracellular recording of local filed potential (LFP) from the iCell cardiomyocytes were performed using the MEA recording system (Muse; Axion BioSystems, Inc.). For co-culture with sympathetic or parasympathetic neurons, day 13 induced cells were directly plated onto the iCell cardiomyocytes with 1:1 mixed medium (iCell maintenance medium: neuron medium).

### Optogenetic stimulation of induced neurons

Humanized channelrhodopsin-2 (ChR2) lentivirus was packaged with pLenti-Synapsin-hChR2(H134R)-EYFP-WPRE construct (Addgene plasmid #20945). Induced neurons were infected with the ChR2 lentivirus. After over one week of infection, the infection efficiency was examined by evaluating YFP expression. To calculate the beating rates of cardiomyocytes co-cultured with induced neurons, movies of phase-contrast image were recorded before and after continuous illumination of blue light (LED-EXTA/GFP, Optocode Corp., Tokyo, Japan) for 20 s. The contraction rates in the movies were analysed using MUSCLEMOTION^46^ (open-source ImageJ plugin)

### Quantification and statistical analysis

All data are expressed as mean ± SD values. Differences between experimental groups were analysed by Student’s *t*-test or Welch’s *t*-test (two groups). Differences among more than two groups were analysed via one-way ANOVA, and Tukey’s post hoc method was used for multiple comparisons. Differences with *P* < 0.05 were considered statistically significant. For GO analysis, GO terms with Benjamini-adjusted *P*-values <0.05 were considered statistically significant.

## Supplementary information

Supplementary Information.

Supplementary List.

Supplementary Movie 1.

Supplementary Movie 2.

Supplementary Movie 3.

Supplementary Movie 4.

Supplementary Movie 5.

Supplementary Movie 6.

## Data Availability

Bulk RNA sequencing data are deposited on DDBJ under accession number: DRA008963. Single-cell RNA sequencing data have been deposited on DDBJ under accession number: DRA009071. All other data that support the findings of this study are available from the corresponding authors on request.
