# Supplementary material for: Selective Induction of Human Autonomic Neurons Enables Precise Control of Cardiomyocyte Beating
Source: Sci Rep. 2020 Jun 11;10:9464. doi: 10.1038/s41598-020-66303-3 (PMC7289887; doi:10.1038/s41598-020-66303-3)
Supplement: Supplementary file 1 — Supplementary Information. [file 41598_2020_66303_MOESM1_ESM.pdf]

Supplementary Information

Selective Induction of Human Autonomic Neurons Enables Precise Control of Cardiomyocyte Beating

Yuzo Takayama[1], Hiroko Kushige[1], Yuka Akagi[1,2], Yutaka Suzuki[3], Yutaro Kumagai[1,2] and Yasuyuki S. Kida[1,2]*

[1]Cellular and Molecular Biotechnology Research Institute, National Institute of Advanced Industrial Science and Technology (AIST), Central 5-41, Higashi 1-1-1, Tsukuba 305-8565 Ibaraki, Japan.

[2]Advanced Photonics and Biosensing Open Innovation Laboratory, National Institute of Advanced Industrial Science and Technology (AIST), Central 5-41, Higashi 1-1-1, Tsukuba 305-8565 Ibaraki, Japan.

[3]Department of Computational Biology and Medical Sciences, The University of Tokyo, 5-1-5 Kashiwanoha, Kashiwa-shi 277-8562 Chiba, Japan.

*Corresponding Author: y-kida@aist.go.jp

a

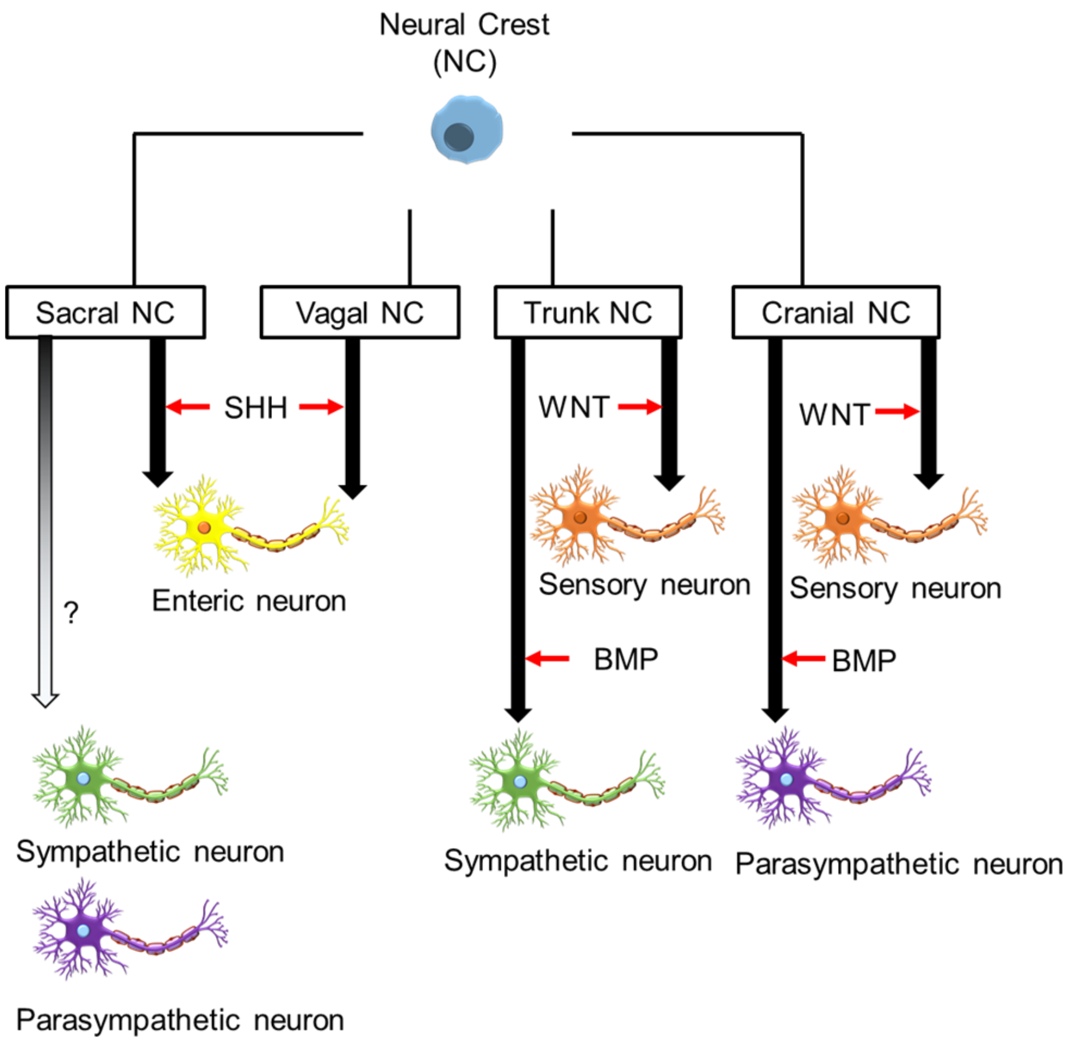

b

GO ID	Term	-log10 (*P*-value)
GO:0006413	translational initiation	2.98
GO:0006412	translation	2.26
GO:0000184	nuclear-transcribed mRNA catabolic process, nonsense-mediated decay	1.96
GO:0006614	SRP-dependent cotranslational protein targeting to membrane	1.60
GO:0000082	G1/S transition of mitotic cell cycle	1.40
GO:0007062	sister chromatid cohesion	1.44

c

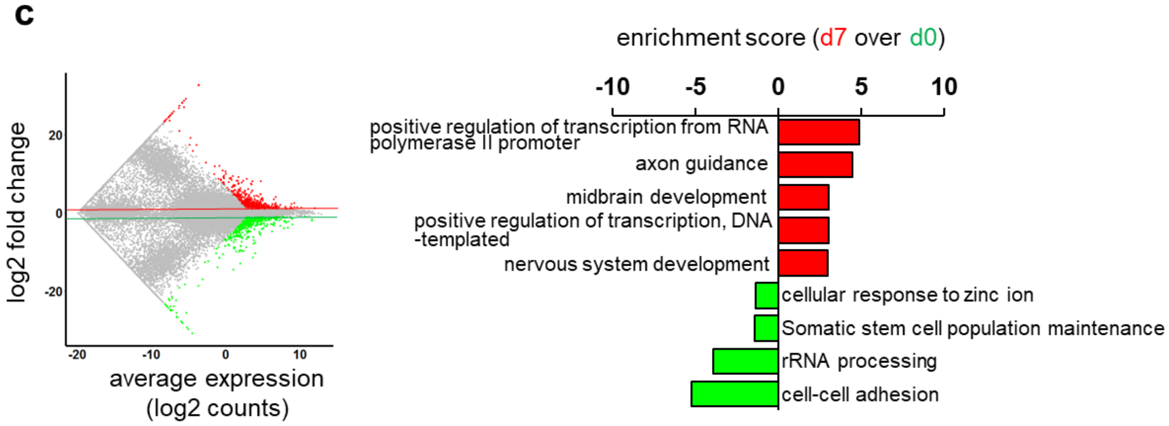
log2 fold change
20
0
-20
-20
-10
0
10
average expression
(log2 counts)
enrichment score (d7 over d0)
-10
-5
0
5
10
positive regulation of transcription from RNA polymerase II promoter
axon guidance
midbrain development
positive regulation of transcription, DNA -templated
nervous system development
cellular response to zinc ion
Somatic stem cell population maintenance
rRNA processing
cell-cell adhesion

d

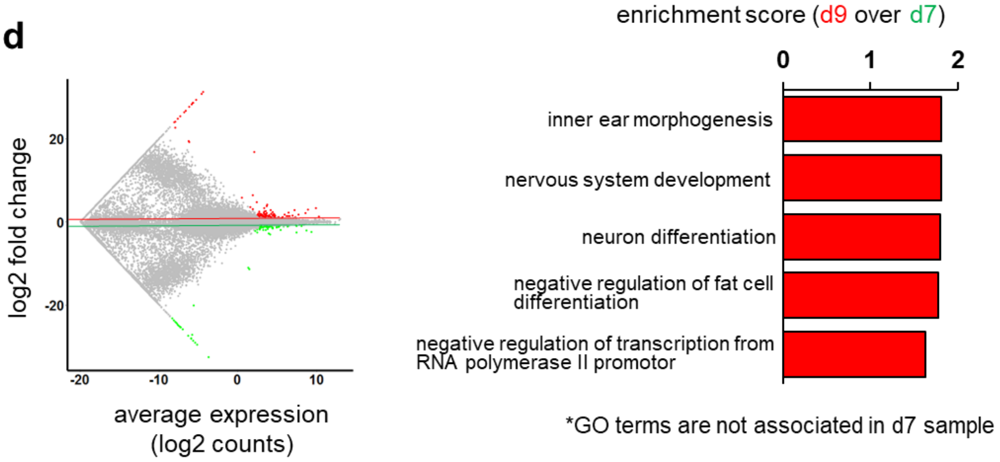
log2 fold change
20
0
-20
-20
-10
0
10
average expression
(log2 counts)
enrichment score (d9 over d7)
0
1
2
inner ear morphogenesis
nervous system development
neuron differentiation
negative regulation of fat cell differentiation
negative regulation of transcription from RNA polymerase II promotor
*GO terms are not associated in d7 sample

e

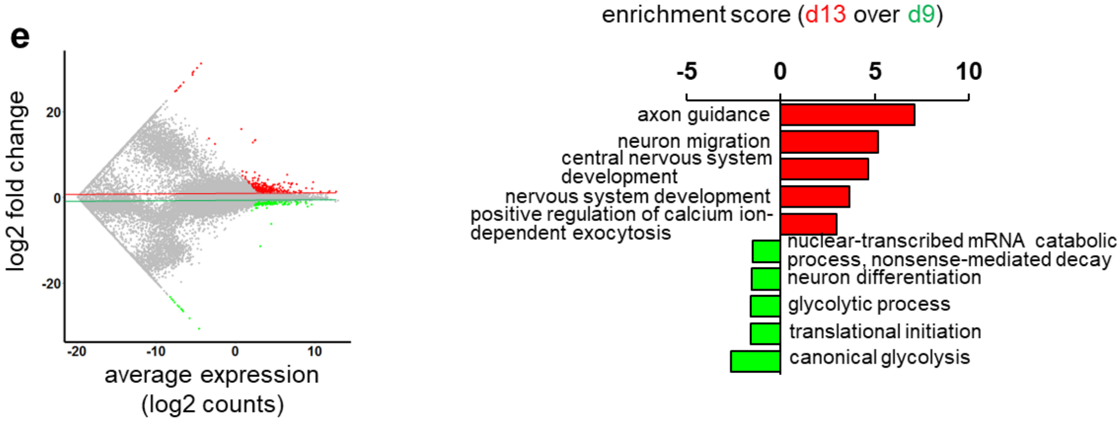
log2 fold change
20
0
-20
-20
-10
0
10
average expression
(log2 counts)
enrichment score (d13 over d9)
-5
0
5
10
axon guidance
neuron migration
central nervous system development
nervous system development
positive regulation of calcium ion-dependent exocytosis
nuclear-transcribed mRNA catabolic process, nonsense-mediated decay
neuron differentiation
glycolytic process
translational initiation
canonical glycolysis

f

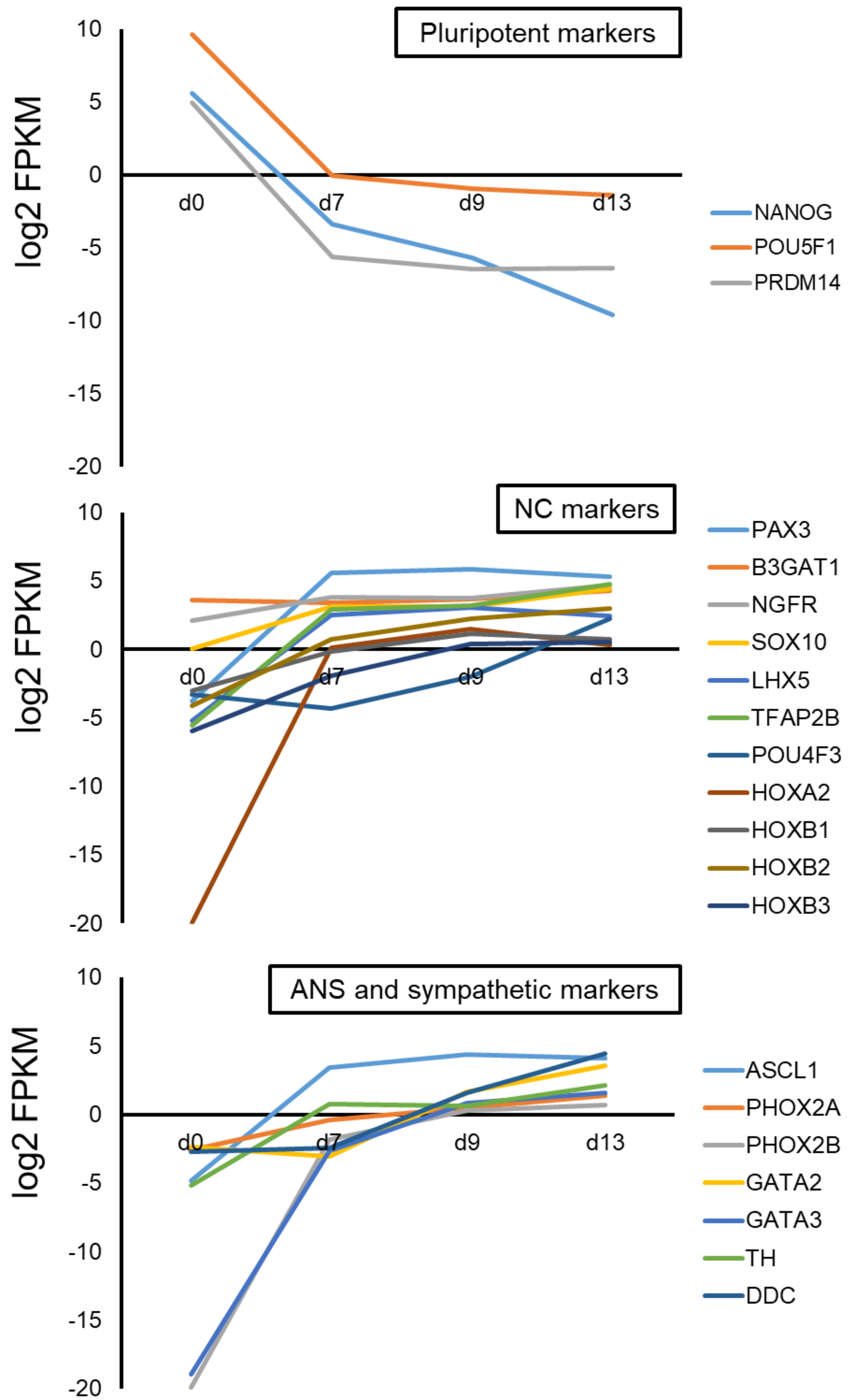
Pluripotent markers
log2 FPKM
10
5
0
-5
-10
-15
-20
d0
d7
d9
d13
NANOG
POU5F1
PRDM14
NC markers
log2 FPKM
10
5
0
-5
-10
-15
-20
d0
d7
d9
d13
PAX3
B3GAT1
NGFR
SOX10
LHX5
TFAP2B
POU4F3
HOXA2
HOXB1
HOXB2
HOXB3
ANS and sympathetic markers
log2 FPKM
10
5
0
-5
-10
-15
-20
d0
d7
d9
d13
ASCL1
PHOX2A
PHOX2B
GATA2
GATA3
TH
DDC

g

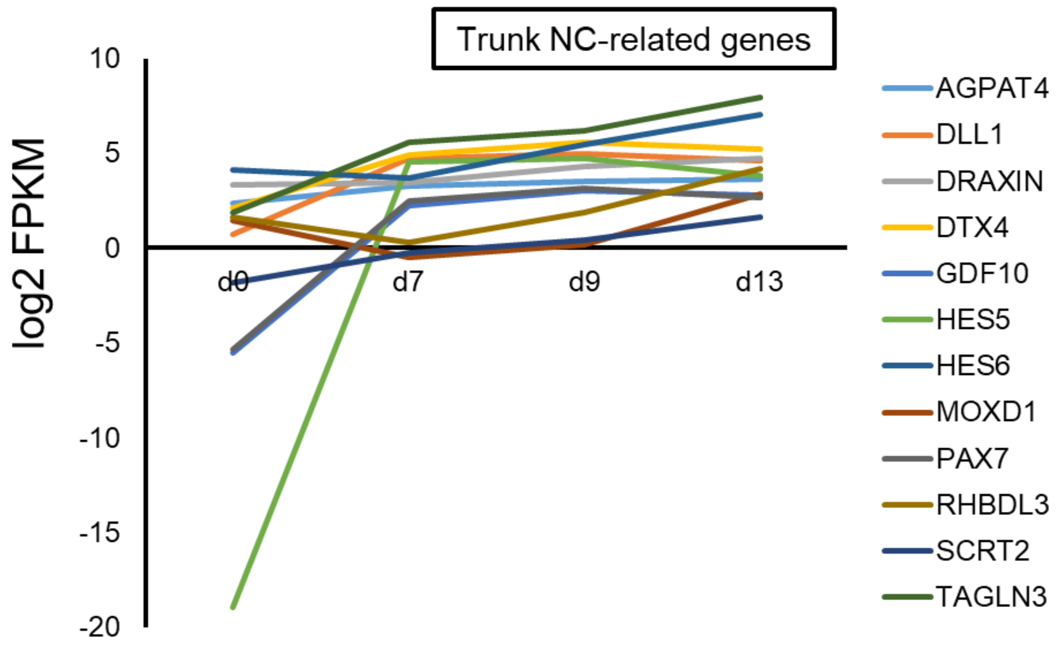

Supplementary Fig. 1.

Related to Fig. 1.

The stepwise autonomic specification method promotes neuronal differentiation in comparison with the conventional neural crest induction method.

a, Schematic representation of the differentiation pathways from NC subtypes and subsequent PNS neuronal subtypes. Sensory neurons are majorly induced from cranial and trunk NC cells with the effects of WNT activation [15,16]. Enteric neurons are majorly induced from vagal and sacral NC cells with the effect of SHH activation [17]. Sympathetic neurons are majorly induced from trunk NC cells, whereas parasympathetic neurons are majorly induced from cranial NC cells. Induction of both sympathetic and parasympathetic neurons was promoted by the effects of BMP activation. **b**, Gene Ontology (GO) analysis for biological processes of genes enriched in cNC-induced cells on day 13. **c-e**, Pairwise comparisons of RNA-seq data in aNC-induced cells between day 7 and 0 (**c**), day 9 and 7 (**d**), and day 13 and 9 (**e**) revealed differentially expressed genes as MA plots. Selected GO terms for biological processes of enriched genes are also shown. GO term selection was based on *P*-values after Benjamini correction lower than 0.05. **f**, Developmental changes in FPKM values of selected marker genes. Genes were considered to be expressed when the value of log2 FPKM was larger than 0. **g,** Developmental changes in FPKM values of selected trunk NC marker genes. Genes were considered to be expressed when the value of log2 FPKM was larger than 0.

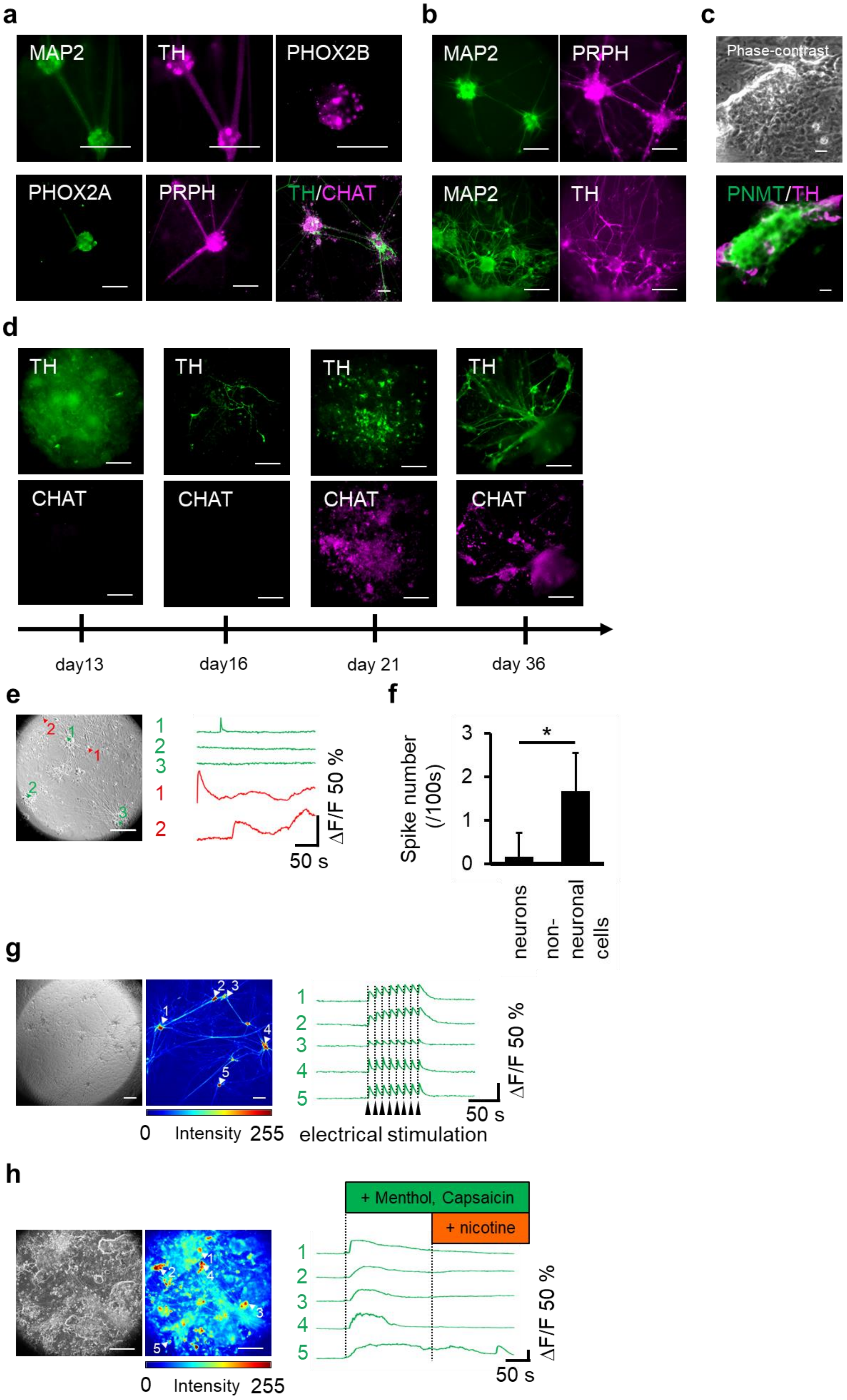
a
MAP2
TH
PHOX2B
PHOX2A
PRPH
TH/CHAT
b
MAP2
PRPH
MAP2
TH
c
Phase-contrast
PNMT/TH
d
TH
CHAT
day13
day16
day 21
day 36
e
ΔF/F 50 %
50 s
f
Spike number (/100s)
neurons
non-neuronal cells
g
Intensity
electrical stimulation
ΔF/F 50 %
50 s
h
+ Menthol, Capsaicin
+ nicotine
Intensity
ΔF/F 50 %
50 s

Supplementary Fig. 2.

Related to Fig. 2.

Stepwise autonomic specification methods applicable for various human pluripotent stem cell (hPSC) lines.

a, Immunostaining of human ESC (H1 line)-derived neurons for MAP2, PRPH, PHOX2A, PHOX2B, TH, and CHAT. Scale bar; 100 μm. **b**, Immunostaining of human iPSC (253G1 line)-derived neurons for MAP2, PERIPHERIN, and TH. Scale bar; 100 μm. **c**, PNMT-positive chromaffin cells emerged in the induced cells. Some PNMT-positive cells were positive for TH. Scale bar; 100 μm. **d**, Immunostaining results of neurons for TH and CHAT at day 13, 16, 21 and 36. Scale bar; 100 μm. **e**, Typical traces of calcium transients of induced neurons (green) or non-neuronal cells (red) indicated in the phase-contrast image on day 45. Neurons were selected within the ganglion-like structures. We measured spontaneous activity for 150 s. **f**, Averaged calcium spike numbers between neurons and non-neuronal cells (n = 25 cells for iNS; n = 23 cells for non-iNs; error bar shows SDs; Welch's t-test $^*P < 0.001$). **g**, Typical traces of calcium transients of induced neurons on day 49. Calcium signals after electrical stimulation were normalized to the signals before stimulation (middle panel). The colour bar shows the fluorescent intensity. Right panel shows representative traces of calcium transients in 5 neurons indicated in the middle panel for 150 s. **h**, Calcium transients of cNC-induced neurons with indicated drugs. Calcium signals after menthol and capsaicin application were normalized to the signals before application (middle panel). The right panel shows representative traces of calcium transients in five cells as shown in the phase-contrast and fluorescent image for 350 s (see also Supplementary Movie 3). The colour bar shows the fluorescent intensity. Scale bar; 100 μm.

a

The top ten enriched genes in cluster A

Gene	log2(fold change)	-log10(*P*-value)
TXNRD2	3.47	30.92
PCP4	2.85	22.30
MIR155HG	3.39	20.49
FOLR1	2.65	18.91
KCNK1	2.60	18.62
DMKN	2.48	18.25
RARRES2	2.24	14.77
MIR222HG	2.25	14.53
CRABP2	2.15	14.31
GS1-600G8.5	2.78	13.68

The GO term enriched in cluster A (Benjamini *P*-value < 0.05)

GO ID	Term	-log10 (*P*-value)
GO:0006364	rRNA processing	1.58

The top ten enriched genes in cluster B

Gene	log2(fold change)	-log10(*P-value*)
CTHRC1	4.63	41.94
TGFBI	5.94	40.76
NNMT	5.35	38.86
COL1A2	4.24	37.25
COL3A1	5.93	37.22
CXCL8	6.75	37.00
COL1A1	4.47	34.80
MFAP4	4.12	32.70
ELN	4.55	32.40
IGFBP4	4.27	31.68

The GO terms enriched in cluster B (Benjamini *P*-value < 0.05)

GO ID	Term	-log10 (*P*-value)
GO:0006614	SRP-dependent cotranslational protein targeting to membrane	12.45
GO:0036498	IRE1-mediated unfolded protein response	8.89
GO:0006413	translational initiation	7.65
GO:0019083	viral transcription	7.23
GO:0000184	nuclear-transcribed mRNA catabolic process, nonsense-mediated decay	6.93
GO:0006412	translation	6.57
GO:0006364	rRNA processing	5.63
GO:0034976	response to endoplasmic reticulum stress	3.77
GO:0071230	cellular response to amino acid stimulus	2.49
GO:0030198	extracellular matrix organization	2.08

The top ten enriched genes in cluster C

Gene	log2(fold change)	-log10(*P*-value)
MT-ND3	2.14	6.60
MT-ATP6	2.12	6.53
MT-CO3	2.09	6.42
MT-ND2	2.07	6.42
MT-CO1	2.04	6.27
MT-ND4L	2.04	6.22
MTRNR2L12	2.11	5.94
MT-ND5	2.00	5.94
MT-CYB	1.99	5.94
MT-ND6	2.01	5.85

The top ten enriched genes in cluster D

Gene	log2(fold change)	-log10(*P*-value)
GPM6A	5.00	16.53
LHX9	7.61	16.53
FXYD6	4.57	15.72
DUSP26	4.91	15.03
FAM57B	4.55	14.84
CAMK2N1	4.96	14.84
UNC5B-AS1	5.62	12.15
TH	6.58	11.97
PODXL2	3.94	11.91
NREP	4.40	11.91

The GO terms enriched in cluster D (Benjamini *P*-value < 0.05)

GO ID	Term	-log10 (*P*-value)
GO:0007399	nervous system development	7.95
GO:0007411	axon guidance	3.02
GO:0007269	neurotransmitter secretion	3.11
GO:0000226	microtubule cytoskeleton organization	3.14
GO:0007264	small GTPase mediated signal transduction	3.13
GO:0007017	microtubule-based process	3.21
GO:0048813	dendrite morphogenesis	3.15
GO:0021762	substantia nigra development	2.86
GO:0007018	microtubule-based movement	2.78
GO:0000086	G2/M transition of mitotic cell cycle	2.39

The top ten enriched genes in cluster E

Gene	log2(fold change)	-log10(*P*-value)
TBX3	6.83	31.85
CNTN1	6.62	31.07
PLEKHD1	6.61	30.14
KCTD16	6.20	29.93
ALDOC	5.61	29.93
NEFH	6.81	29.84
NRCAM	5.76	29.74
ELAVL2	5.16	29.68
GABRG2	5.56	29.55
SYT2	7.15	29.38

The GO terms enriched in cluster E (Benjamini *P*-value < 0.05)

GO ID	Term	-log10 (*P*-value)
GO:0007399	nervous system development	7.32
GO:0007269	neurotransmitter secretion	5.84
GO:0000226	microtubule cytoskeleton organization	5.58
GO:0019886	antigen processing and presentation of exogenous peptide antigen via MHC class II	5.47
GO:0007411	axon guidance	4.80
GO:0016079	synaptic vesicle exocytosis	4.31
GO:0007018	microtubule-based movement	4.08
GO:0016241	regulation of macroautophagy	4.06
GO:0045773	positive regulation of axon extension	3.75
GO:0006695	cholesterol biosynthetic process	3.36

b

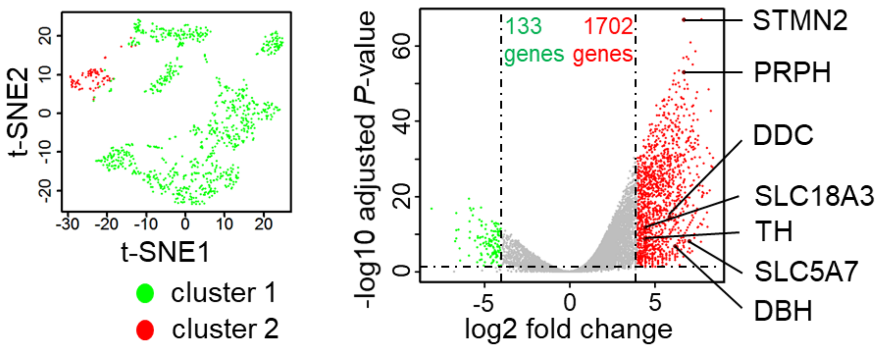

c

The top ten enriched genes in cluster 1

Gene	log2 fold change	-log10 adjusted *P*-value
IGFBP7	5.92	19.42
SPARC	5.20	17.06
FBLN1	5.82	16.93
FOLR1	8.11	16.80
TTR	5.65	16.80
CYR61	5.78	16.36
SOD3	6.65	15.57
GDF15	5.69	15.01
AXL	5.30	13.78
CTGF	5.88	13.38

The top ten enriched genes in cluster 2

Gene	log2 fold change	-log10 adjusted *P*-value
DCX	7.75	67.09
STMN2	6.71	66.86
NEFL	7.11	60.90
ELAVL4	7.40	58.51
MAPT	6.89	57.08
GNAO1	6.70	56.69
HMP19	6.99	56.43
SCG3	7.24	55.07
OLFM1	6.43	53.32
PRPH	6.72	52.88

Supplementary Fig. 3.

Related to Fig. 3.

Single-cell RNA-seq revealed distinct populations of human pluripotent stem cell (hPSC)-derived autonomic nervous system (ANS) neurons.

a, Top ten enriched genes and Gene Ontology terms for biological process in clusters A–E. **b**, Left, t-SNE visualization based on K-means clustering (k = 2) result in clustering non-neuronal (cluster 1, green, n = 906) and neuronal (cluster 2, red, n = 87) populations; right, pairwise comparison of neuronal vs non-neuronal populations shows differentially expressed genes as a volcano plot (green genes, cluster 1 enriched; red genes, cluster 2 enriched; fold-change ≥ |16|, adjusted P < 0.05). Peripheral neuron markers (STMN2 and PRPH), sympathetic neuron markers (TH, DDC, and DBH), and parasympathetic neuron markers (SLC5A7 and SLC18A3) are identified in red genes. **c**, The top ten enriched genes based on the adjusted P-value in clusters 1 and 2. Peripheral neuron markers (STMN2 and PRPH) were identified in cluster 2 and are highlighted in yellow.

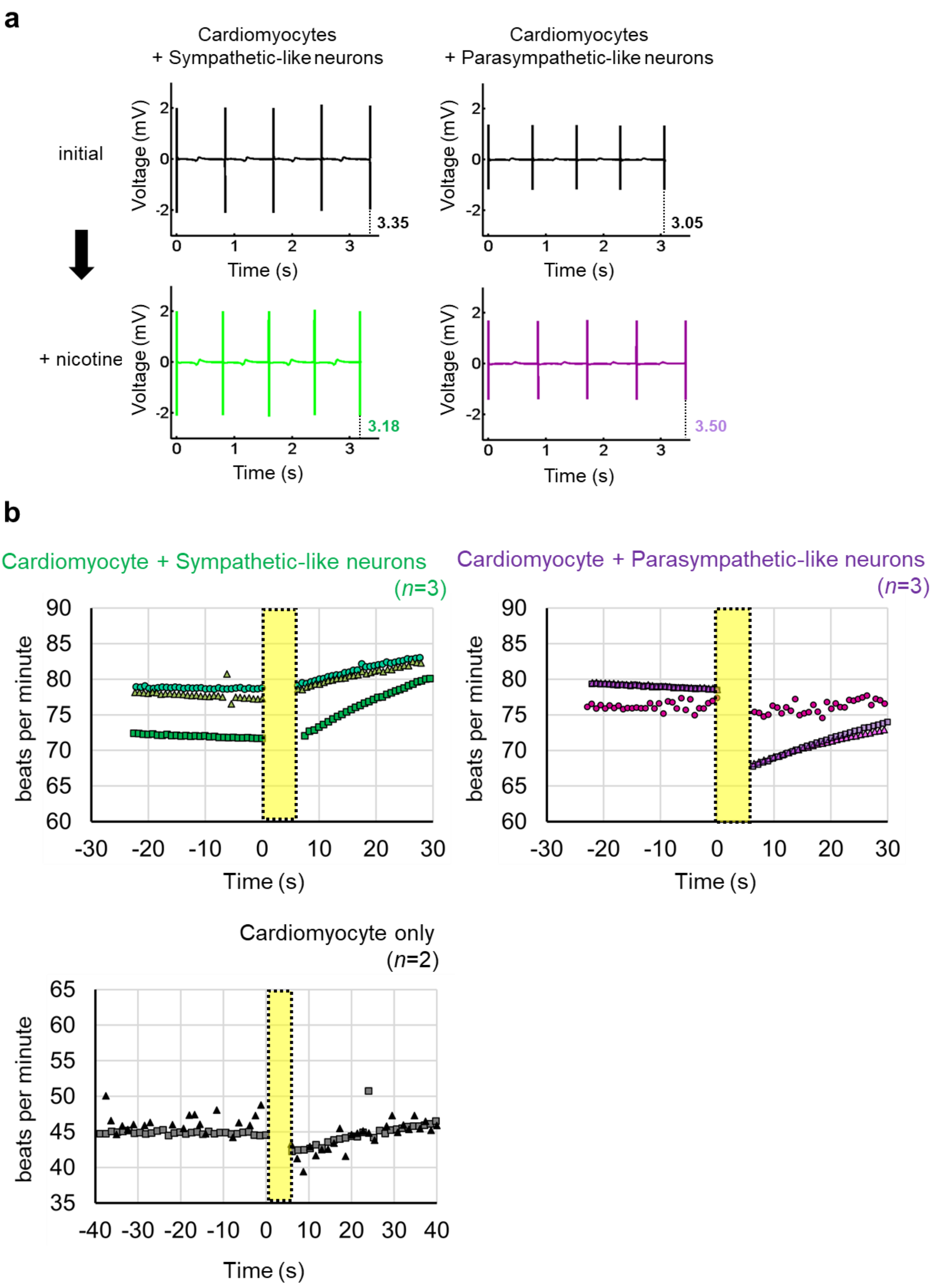
a
Cardiomyocytes
+ Sympathetic-like neurons
Cardiomyocytes
+ Parasympathetic-like neurons
initial
+ nicotine
Voltage (mV)
Time (s)
3.35
3.05
3.18
3.50
b
Cardiomyocyte + Sympathetic-like neurons
(n=3)
Cardiomyocyte + Parasympathetic-like neurons
(n=3)
beats per minute
Time (s)
Cardiomyocyte only
(n=2)

Supplementary Fig. 4.

Related to Fig. 4.

Activation of human pluripotent stem cell (hPSC)-derived sympathetic-like and parasympathetic-like neurons modulate spontaneous beating of human induced PSC (hiPSC)-derived cardiomyocytes.

a, Left, application of 20 μM nicotine increased the beating rate of cardiomyocytes co-cultured with sympathetic-like neurons; right, application of 20 μM nicotine decreased the beating rate of cardiomyocytes co-cultured with parasympathetic-like neurons. Representative traces of local field potential (LFP) recorded in a single electrode site for duration of 5 LFPs before and after nicotine application are shown. **b**, Beat frequency changes in hiPSC-derived cardiomyocytes immediately before and after 20 μM nicotine stimulation. Nicotine was applied to the sample at 0 s. Each point was calculated from the interval between two LFPs and points from 30 LFPs for both before and after nicotine application are shown. Each trace indicates a different sample (*n* = 3, cardiomyocyte-sympathetic co-culture sample; *n* = 3, cardiomyocyte-parasympathetic co-culture sample; *n* = 2, cardiomyocyte culture sample). In the yellow window, LFPs were not recorded because of artifacts in nicotine application. Stimulation of sympathetic-like neurons transiently increased the beating rate of hiPSC-derived cardiomyocytes and parasympathetic-like neurons decreased their beating rate. In contrast, in the cardiomyocyte only culture sample, nicotine application induced slight changes in the beating rate.

Supplementary List

All enriched genes of five clusters in single-cell RNA-seq data. Genes were considered to be expressed when UMI count > 1, UMI count -fold change >2, and adjusted *P*-value < 0.05.

Supplementary Movie 1.

Time-lapse images of Synapsin-1-GFP-infected neurons, Related to Fig. 2.

The movie shows phase-contrast and GFP-fluorescence images of neurons during days 19–22. Images are captured every 30 min.

Supplementary Movie 2.

Pharmacological evaluation of aNC-induced neurons, Related to Fig. 2.

The movie shows calcium imaging of neurons exposed to 10 μM capsaicin, 100 μM menthol, and 40 μM nicotine with aNC method. Calcium signals are drastically increased upon nicotine application. The observation period was 350 s.

Supplementary Movie 3.

Pharmacological evaluation of cNC-induced neurons, Related to Fig. 2.

The movie shows calcium imaging of neurons exposed to 10 μM capsaicin, 100 μM menthol, and 40 μM nicotine with cNC method. Calcium signals are drastically increased upon menthol and capsaicin application. The observation period was 350 s.

Supplementary Movie 4.

Response of hPSC-derived sympathetic-like neurons to 6-OHDA, Related to Fig. 3.

The movie shows 100 μM 6-OHDA-treated sympathetic-like neurons for approximately 20 h (5-min intervals). Neurons were labelled with Synapsin-1-GFP vector for visualization.

Supplementary Movie 5.

Response of human pluripotent stem cell (hPSC)-derived parasympathetic-like neurons to 6-OHDA, Related to Fig. 3.

The movie shows 100 µM 6-OHDA-treated parasympathetic-like neurons for approximately 20 h (5-min interval). Neurons were labelled with Synapsin-1-GFP vector for visualization.

Supplementary Movie 6.

Photoactivation of ChR2-expressing sympathetic-like neurons increases the beating rates of human induced PSC (hiPSC)-derived cardiomyocytes, Related to Fig. 4.

The movie shows that blue-light stimulation of ChR2-expressing hPSC-derived sympathetic-like neurons results in increased beating rates of co-cultured hiPSC-derived cardiomyocytes.
